# What predicts persisting social impairment following pediatric traumatic brain injury: contribution of a biopsychosocial approach

**DOI:** 10.1017/S0033291722000186

**Published:** 2023-06

**Authors:** Vicki Anderson, Stephen J. C. Hearps, Cathy Catroppa, Miriam H. Beauchamp, Nicholas P. Ryan

**Affiliations:** 1 Murdoch Children's Research Institute, Flemington Road, Parkville, Victoria, 3052, Australia; 2 Royal Children's Hospital, Flemington Road, Parkville, Victoria, 3052, Australia; 3 University of Melbourne, Flemington Road, Parkville, Victoria, 3052, Australia; 4 University of Montreal, P.O. Box 6128, Center-ville branch, Montreal, QC, H3C 317, Canada; 5 St Justine Hospital, Avenue Ellendale, Montreal, QC, Canada; 6 Deakin University, 221 Burwood Highway, Burwood, Australia

**Keywords:** Behavior, brain injury, child, recovery, social cognition, social impairment

## Abstract

**Background:**

Psychosocial deficits, such as emotional, behavioral and social problems, reflect the most common and disabling consequences of pediatric traumatic brain injury (TBI). Their causes and recovery likely differ from physical and cognitive skills, due to disruption to developing brain networks and the influence of the child's environment. Despite increasing recognition of post-injury behavioral and social problems, there exists a paucity of research regarding the incidence of social impairment, and factors predicting risk and resilience in the social domain over time since injury.

**Methods:**

Using a prospective, longitudinal design, and a bio-psychosocial framework, we studied children with TBI (*n* = 107) at baseline (pre-injury function), 6 months, 1 and 2-years post-injury. We assessed intellectual ability, attention/executive function, social cognition, social communication and socio-emotional function. Children underwent structural magnetic resonance imaging (MRI) at 2–8 weeks post-injury. Parents rated their child's socio-emotional function and their own mental health, family function and perceived burden.

**Results:**

We distinguished five social recovery profiles, characterized by a complex interplay between environment and pre- and post-TBI factors, with injury factors playing a lesser role. Resilience in social competence was linked to intact family and parent function, intact pre-injury adaptive abilities, post-TBI cognition and social participation. Vulnerability in the social domain was related to poor pre- and post-injury adaptive abilities, greater behavioral concerns, and poorer pre- and post-injury parent health and family function.

**Conclusions:**

We identified five distinct social recovery trajectories post-child-TBI, each characterized by a unique biopsychosocial profile, highlighting the importance of comprehensive social assessment and understanding of factors contributing to social impairment, to target resources and interventions to children at highest risk.

The manner in which a child operates within their social environment is critical for forming lasting relationships, community participation and quality of life (Blakemore, 2008; Cacioppo, 2002). What appears automatic is, in fact, a highly complex**,** dynamic interplay between developing neural networks (social brain network) and environmental influences (Adolphs, 2009; Adolphs & Anderson, 2013; Belsky & de Haan, 2011).

Pediatric traumatic brain injury (TBI) has been linked to an elevated risk of social impairment, although mechanisms underpinning this risk are unclear, as are factors that can promote resilience. Compared to their healthy peers, children with TBI have lower self-esteem, fewer friendships, more aggressive and antisocial behaviors, and poorer emotional and behavioral regulation (Andrews, Rose, & Johnson, 1998). Some experience increasing social impairments with time post-injury, regardless of injury severity (Anderson et al., 2017; Ryan et al., 2021a; Yeates, Taylor, Walz, Stancin, & Wade, 2010), with adolescents with TBI having a three-fold greater risk of internalizing and externalizing behaviors than healthy peers (Max et al., 1998).

The mechanisms underpinning social impairment after pediatric TBI may be best represented by a biopsychosocial framework. For example, Yeates et al. (2007) propose that, post-TBI, social skills are susceptible to both injury (injury type, severity, nature, extent of brain pathology) and non-injury factors [parenting style, family function, socio-economic status (SES)]. We have proposed and recently validated a similar framework (SOCIAL: Figure 1: Beauchamp and Anderson, 2010; Tuerk, Anderson, Bernier, and Beauchamp, 2021) encompassing both typical and atypical social function, highlighting the mediating role of the brain (development, integrity) and environment (social status, family and parent function) and the contribution of child factors to social competence.

**Fig. 1.**
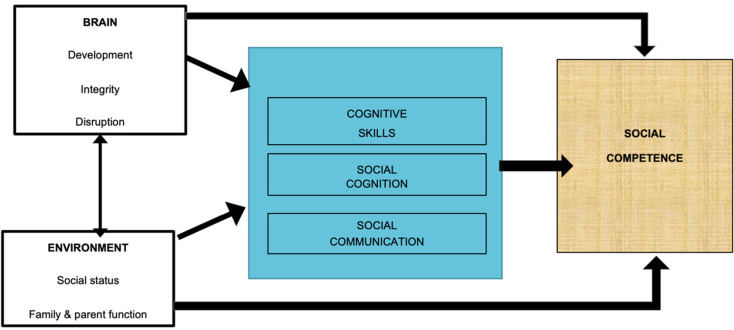
The SOCIAL model, adapted from Beauchamp & Anderson, Psychological Bulletin, 2010.

Growing evidence supports the importance of the integrity of the *social brain network* for intact social functions, including emotion recognition (Blakemore & Choudhury, 2006; Ryan et al., 2013), theory of mind (Robinson et al., 2014; Ryan et al., 2015, 2016a, 2017a, 2017b; Turkstra, Dixon, & Baker, 2004; Yeates, 2013), pragmatic language (Ryan et al., 2013, 2018), and social problem solving (Beauchamp, Dooley, & Anderson, 2013; Janusz, Kirkwood, Yeates, & Taylor, 2002; Ryan et al., 2021b). The social brain network develops and becomes refined through childhood and adolescence (Beauchamp & Anderson, 2010). It comprises numerous brain regions vulnerable to the diffuse axonal injury characteristic of TBI, for example, aspects of the prefrontal cortex, temporo-parietal junction, insula, and amygdala (Adolphs, 2001, 2003). TBI during childhood has the potential to derail the maturation of this network resulting in social dysfunction (Anderson, Spencer-Smith, & Wood, 2011; Zhi, Ryan, Konjarski, Catroppa, & Stargatt, 2021).

The importance of the child's *environment* is well established in the developmental psychology literature, with SES, quality of the home and access to resources correlated with children's social outcomes (Belsky & de Haan, 2011; Bowlby, 1962; Masten et al., 1999). There is growing evidence for the role of these distal influences as well as more proximal factors (family function, parent mental health) for child recovery following TBI (Anderson, Catroppa, Haritou, Morse, & Rosenfeld, 2005; Beauchamp, Seguin, Gagner, Lalonde, & Bernier, 2020; Chavez-Arana et al., 2019; Ryan et al., 2016b; Wade et al., 2011; Woods, Catroppa, Barnett, & Anderson, 2011).

The SOCIAL model also emphasizes the contribution of *cognitive skills* to social competence. Deficits in the attention-executive dimension (e.g. dysregulation) have been linked to negative social outcomes: aggression, delinquency, antisocial behavior and inappropriate social interactions (Barkley, Edwards, Laneri, Fletcher, & Metevia, 2001; Crick & Dodge, 1994; Lemerise & Arsenio, 2000; Ryan et al., 2021a). Further, in everyday interactions, slowed cognition can hinder a child's ability to follow a conversation or keep pace with processing demands (Anderson, 2002). Similarly, social aspects of communication, including joint attention and integration of affect and gesture with communication, are directly linked to successful relationships and peer acceptance (Beauchamp & Anderson, 2010; Turkstra et al., 2004).

Despite a growing literature implicating *brain, environment and cognition* in social impairment post pediatric TBI, many gaps remain. Few studies have examined these dimensions simultaneously, leading to a poor understanding of their independent and cumulative contributions to the outcome. Reliance on group means has further limited accuracy of data regarding the frequency of social impairment. Finally, most studies employ a ‘risk’ perspective, missing the opportunity to identify modifiable protective factors, which may guide intervention, promote ‘*resilience*’ and optimize child outcomes (e.g. family factors). Resilience is broadly defined as the achievement of a good outcome, despite challenging circumstances (e.g. TBI) (Masten & Cicchetti, 2012). An accumulation of risks and stressors, such as those present following TBI, and interactions among these, may inhibit resilience, causing adverse outcomes (Smith & Carlson, 1997). Conversely, protective factors (e.g. environment, family function, intervention) provide opportunities to support resilience (Anderson et al., 2020; Holland & Schmidt, 2015).

To extend current knowledge, we employed a bio-psycho-social framework and aimed to identify: (i) the incidence of social impairment over the 2 years post-TBI, (ii) specific social recovery trajectories and (iii) factors characterizing these trajectories. We expected to find multiple social recovery trajectories, differentiated by varying patterns of brain, environmental and cognitive risk and protective factors.

## Materials and methods

### Design

We employed a single site, prospective, longitudinal case–control design to study children's social function following TBI: pre-injury (T0), 6 months (T1), 1 year (T2) and 2 years (T3) post-injury. While the study included a typically developing comparison group (TDC) (see Anderson et al., 2017), only children with TBI were included in the trajectory analyses.

### Participants

This study comprised 107 children (74 males) with TBI, representing 78.7% of the original sample, recruited at the time of injury. Children were recruited from consecutive presentations to The Royal Children's Hospital (RCH), a statewide tertiary hospital based in Melbourne, Australia.

Inclusion criteria were: (i) 5–16 years at recruitment; (ii) evidence of closed head injury (e.g. blow to head), including a period of altered consciousness, or at least 2 post-concussive symptoms; (iii) sufficient information to determine injury severity [i.e. Glasgow Coma Scale (GCS: Teasdale and Jennett, 2004)], neurological and radiological findings; (iv) no pre-injury neurological or developmental disorder, (v) no inflicted injury, or previous TBI; (vi) no pre-injury diagnosis of, or intervention for, social problems; (vi) English speaking; and (vii) completion of acute MRI, and 6, 12 and 24-month assessments.

TBI severity was categorized as: *mild* (*n* = 68): GCS 13–15 on hospital presentation, no intracranial pathology on MRI and/or skull fracture not requiring surgical intervention; *moderate-severe* (*n* = 39): GCS 3–12 on hospital presentation, and/or evidence of intracranial pathology on MRI scan, skull fracture requiring surgical intervention. Children with TBI received routine post-TBI care.

### Measures

We selected the measures below to provide quantification of the core components of the SOCIAL model (Fig. 1): brain, environment and cognitive skills. Study measures are detailed in Table 1. For measures without normative data, results from the larger study's typically developing control group (TDC: *n* = 39) are used for comparison (Anderson et al., 2013, 2017). For parent questionnaires, information was provided primarily by mothers (84.2%) with 14.0% completed by fathers, 0.9% by both parents and 0.9% classified as ‘other’ (grandparent/guardian).

**Table 1.** Assessment measures and follow-up time points

Test measure	Baseline T0	6 Months T1	1 Year T2	2 Years T3
Injury/ demographic data	X			
Primary outcome ABAS Social	X*	X	X	X
Brain structure: MRI scan	X			
Parent status				
Family function (FAD)	X*	X	X	X
Mental Health (GHQ)	X*	X	X	X
Burden (FBII)		X		X
*Child measures*				
Descriptors				
Adaptive behavior (ABAS GAC)	X*	X	X	X
IQ (WASI-2)		X		X
Social Model measures: child direct				
Processing Speed (PSI)		X		X
Working Memory: Digit span (DS)		X		X
Attention & EF (CC, WDW, SSDT)		X		X
Social Cognition (EEFT)		X		X
Social Communication (TLC: MI)		X		X
SOCIAL Model measures*:* parent report of child behavior				
Behavior (CBCL)	X*	X	X	X
Social participation (CASP)	X*	X	X	X
Executive function (BRIEF GEC)	X*	X	X	X

X, Assessment included in this paper.

*Parents provided retrospective ratings of pre-injury functioning at the time of injury.

AUSE106, Australian Socioeconomic Index 2006; ABAS Social, Adaptive Behavior Assessment System Social Scale; WASI, Wechsler Abbreviated Intelligence Scale; PSI, Processing Speed Index; DS, Digit Span; CC, Creature Counting; WDW, Walk Don't Walk; SSDT, Sky Search Dual Task, TLC:MI, Test of Language Competence Making Inferences; EEFT, Emotive and Emotional Faces; ABAS GAC, Adaptive Behavior Assessment System Global Adaptive Composite; CBCL, Child Behavior Checklist; CASP, Child and Adolescent Scale of Participation; BRIEF GEC, Behavioral Rating Inventory of Executive Function Global Executive Composite; FAD, Family Assessment Device; GHQ, General Health Questionnaire; FBII, Family Burden of Injury Inventory.

#### Primary outcome: social competence 2 years post-injury

*Social Competence:* Adaptive Behavior Assessment System – II (ABAS: Harrison and Oakland, 2003) is a 232 item, a parent-rated questionnaire assessing children's daily living and coping skills, generating a Global Adaptive Composite (ABAS:GAC) and three domain scores (Conceptual, Social, Practical) (*M* = 100, s.d. = 15). The ABAS:GAC provides a general index of adaptive behavior and was employed as a sample descriptor. In keeping with the study focus on social competence, the ABAS-II Social domain score (ABAS: SOC: T0, T1, T2, T3) was the primary outcome for the study. It targets interpersonal and social competence skills, incorporating the 45 items of the Social and Leisure skill areas and has been shown to be sensitive to the social difficulties demonstrated by children with TBI (Anderson et al., 2017): skills needed to interact socially (e.g. *‘initiate and maintain friendships’*), get along with other people including having friends (*‘keeps a stable group of friends’*), showing and recognizing emotions (e.g. *‘shows sympathy for others when they are sad or upset’*), assisting others (*e.g. ‘offers assistance to others’*), and understanding and applying social rules (*e.g. ‘Says ‘thank-you’ when given a gift’*). As described in the ABAS-II manual, scores ⩾ 110 were classified as ‘above average’, 90–109 ‘average’, 80–89 ‘below average’, 70–79 ‘borderline’ and < 70 ‘impaired/extremely low’.

#### Brain (injury characteristics and brain integrity)

*Injury factors:* GCS (lowest score in the 24 h post-injury), coma duration, surgical intervention, and cause of injury were recorded.

*Brain structure* (*MRI scan*)*:* Children underwent a MRI brain scan 2–8 weeks post-injury. MR images were acquired on a 3 Tesla Siemens Trio scanner (Siemens Medical Systems, Erlangen, Germany) fitted with a 32-Channel matrix head coil. Conventional MR sequences were performed using a standardized imaging protocol that included a susceptibility-weighted imaging (SWI) sequence, which is a variant of the standard 3D FLASH sequence that exploits the signal loss from shortened T2* characteristics of calcium- and deoxyhemoglobin-containing lesions (see Beauchamp et al., 2011). The images are T2* weighted because of the range of acceptable TEs used in the acquisition (18–22 ms). Increased sensitivity to shortened T2* lesions is due to the image reconstruction methods employed. The magnitude and phase images are reconstructed from the data set. Phase images display a high sensitivity to local susceptibility variations and are used as an image mask to be combined with the magnitude data set. The combined data set is then reconstructed using a sliding window (eight individual slices compressed into one image), minimum intensity projection (MIP) data set. The total acquisition time for the MRI protocol was 31:53 min.

*Morphometric analyses*: FreeSurfer image analysis suite (http://surfer.nmr.mgh.harvard.edu.au) was used to automatically parcellate cortical gray matter and sub-cortical structure. Using a probabilistic labeling algorithm that relies on gyral and sulcal information, the cortex was parcellated into anatomical regions of interest (ROI) based on the Desikan–Killany atlas (Desikan et al., 2006) and projected back into participants' native space. Gray matter volumes were extracted for each ROI bilaterally and assigned to domain-specific [Mentalizing Network (MN), Mirror Neuron Empathy Network (MNEN)] and domain-general neural network packages [Default Mode Network (DMN), Salience Network (SN), Central Executive Network (CEN)]. ROIs for each network were summed to calculate overall volumes for each of the five neural networks of interest (Ryan et al., 2017a). Detailed procedures for SWI analyses are detailed in Ryan et al., 2015. In brief, SWI sequences were visually inspected, and lesions were coded according to a modified Coffey classification (Beauchamp et al., 2011; Coffey and Fiegel, 1990) which assesses signal abnormality on SWI images. Lesion load was calculated as total number of gray and white matter neuroanatomical regions with SWI abnormality (Kraus et al., 2007).

#### Environment (distal and proximal factors)

*Distal factors: SES*: The Australian Socioeconomic Index 2006 (AUSEI06: McMillan, Beavis, and Jones, 2009) assigns occupational status scores coded in accordance with the official occupational classifications of the Australian Bureau of Statistics (the Australian and New Zealand Standard Classification of Occupations, ANZSCO), with a scale range of 0–100. Lower scores represent lower SES.

*Proximal factors*: (i) *Family function:* Family Assessment Device (FAD: Epstein, Ryan, Bishop, Miller, and Keitner, 2003): 60-item questionnaire rating overall health of the family. The General Function score [reliability = *0.83–0.86:* (Kabacoff, Miller, Bishop, Epstein, and Keitner, 1990) was employed in analyses], and ranges from 1 (healthy) to 4 (unhealthy) (TDC: *M* = 1.48, s.d. = 0.33); (ii) *Parent mental health:* General Health Questionnaire (GHQ-28: Jackson, 2007): 28-item questionnaire rating parents' psychological distress across four domains: somatic symptoms, anxiety/insomnia, social dysfunction and severe depression. The total GHQ Score maximum is 28 (internal consistency = 0.86). A total score of ⩾ 4 indicates psychological distress (TDC: *M* = 1.38, s.d. = 2.99); and (iii) *Family burden:* Family Burden of Injury Inventory (FBII: Burgess et al., 1999) was developed to assess the unique burdens and challenges of pediatric TBI for families. It assesses perceived family burden across 27 items and 5 subscales (Child, Spouse, Others, Siblings, Routines, Overall Stress). The Total score ranges from 0 (not stressful) to 4 (extremely stressful) (TDC: *M* = 0.09, s.d. = 0.28).

#### Child-direct measures

*Descriptive variables:* (i) *Adaptive abilities*: The ABAS:GAC (see above) was used as an index of children's pre-injury ability*;* (*ii*) *Intelligence:* Wechsler Abbreviated Intelligence Scale: 2 subtest form (WASI: Wechsler, 1999) assessed IQ at 6 months post-injury. Full-Scale Intelligence Quotient (FSIQ) (*M* = 100, s.d. = 15) was employed in analyses.

#### SOCIAL model variables

*Attention/Executive function*: (a) speed of processing: Processing Speed Index (PSI: Wechsler, 2003) (*M* = 100, s.d. = 15); (b) attention capacity: Digit Span Test (DST: Wechsler, 2003) (*M* = 10, s.d. = 3); (c) Test of Everyday Attention for Children (Manly, Robertson, Anderson, & Nimmo-Smith, 1999): attentional shift: Creature Counting (CC), inhibitory control: Walk Don't Walk (WDW:) and divided attention: Sky Search Dual Task (SSDT) (*M* = 10, s.d. = 3); (d) *Everyday executive function:* Behavior Rating Inventory of Executive function (BRIEF: Gioia, Isquith, Guy, and Kenworthy, 2000): parent ratings of children's executive function in daily life. The Global Executive Composite (GEC: *M* = 50, s.d. = 10) was used in analyses, with scores 60–64 considered ‘borderline’ and ⩾ 65 ‘impaired’.

*Social Cognition:* (a) Emotional and Emotive Faces (EEFT: Dennis, Barnes, Wilkinson, and Humphreys, 1998); five vignettes assessed emotion identification and theory of mind through basic and complex judgements about real and deceptive emotions. Participants choose from face drawings depicting emotions (happy, yucky, scared, angry, sad, neutral) of different degrees (e.g. very sad, a little sad). Measures were: EEFT: LOOK: emotive communication (i.e. understanding of the emotion the character expresses socially, which may be different from the felt emotion): for example, ‘*How did Terry look on his face when the dog was growling at him?’* (TDC: *M* = 12.42, s.d. = 3.19); and (b) EEFT: FEEL: emotional expression (i.e. understanding of how a character actually feels): for example, ‘*How did Terry feel inside when the dog was growling at him?*’ (TDC: *M* = 8.5, s.d. = 0.94).

*Social Communication* Making Inferences [MI: Test of Language Competence–Expanded Edition (TLC-E: Wigg and Secord, 1989): pragmatic language skills (e.g. make logical inferences) (*M* = 10, s.d. = 3)].

#### Child socio-emotional function

*Child Behavior:* The Child Behavior Checklist (parent) (CBCL: Achenbach & Rescorla, 2001): a 113-item questionnaire rating child behavior (T0, T3). Total, Internalizing and Externalizing scores were derived (*M* = 50, s.d. = 10). Higher scores reflect more behavioral difficulties, with scores > 60 representing significant behavioral concerns.

*Social participation:* Child and Adolescent Scale of Participation (CASP: Bedell, 2008): a parent-rated measure of participation generating a Total Score (range 0–100), Home, Community, School and Activities subscales (TDC: *M* = 99.55, s.d. = 0.88).

### Procedure

The study was approved by The RCH Human Research Ethics Committee and The Victorian Department of Education Research Ethics Committee, Melbourne, Australia. Children were screened for eligibility and recruited immediately post-injury via review of admissions records. *Pre-injury parent ratings were collected on recruitment* (*< 2 weeks post-TBI*). Participating families were then seen at outpatient clinics and children were assessed by trained psychology interns. MRI scans were conducted at 2–8 weeks post-injury (*M* = 39.25, s.d. = 27.64 days). All study measures were administered and scored in accordance with published author guidelines.

### Statistical analysis

Group-based trajectory modeling (GBTM), a specialized form of finite mixture modeling not requiring complete data across all time points (Nagin, 2005) was used to investigate recovery trajectories over 2 years post-TBI. For a hypothesized number of underlying latent groups, it uses maximum likelihood estimation to identify distinctive clusters of individuals following similar trajectories for a nominated outcome, the ‘primary outcome’, outlines the shape of each trajectory, size of each group, and profiles the characteristics of individuals within trajectory groups. All analyses were conducted in STATA/ICv13.1 (StataCorpLLC), and GBTM using the traj plugin. Alpha was set to 0.05.

Model selection comprised two stages (i) identifying an optimal number of trajectory groups; and (ii) determining preferred polynomial orders specifying the shape of identified trajectories. The best-fitting models were determined for two to five groups, and then were compared on Bayesian Information Criterion (lowest), the parsimony of models (log-likelihood), entropy (>0.8), and fit with the prior theory.

Factors relating to trajectory group membership were investigated using analysis of variance (ANOVA) for continuous variables or chi-squared/Fisher's exact test for categorical variables. Bonferroni adjustment pairwise comparisons were carried out between groups.

## Results

### Sample characteristics

The current sample comprised 107 children with TBI, 95.5% of the original sample of 112 (4 withdrew and 1 had insufficient data for trajectory analysis). No differences were detected between participating and non-participating groups for *age at injury*, sex, SES or injury severity. Table 2 details sample demographics and injury characteristics: Two-thirds of the sample were male and 63.55% (*n* = 68) had mild TBI. Sample pre-injury characteristics were within normal expectations for child adaptive skills, social skills, social participation, IQ, executive skills and behavior. Family/parent function (SES, family function, parent mental health) was also within the normal range.

**Table 2.** Sample demographics and injury characteristics

	Statistic
***N***	107
No Males (*n* %)	74 (69.16)
SES *M* (s.d.)	58 (22.5)
Age at Injury *M* (s.d.)	10.4 (2.5) [5.8–14.8]
Cognitive function (IQ, 6 months) *M* (s.d.), [range]*	97.9 (13.7) [61.0–138.0]
Injury severity *n* (%)	
Mild	68 (63.55)
Moderate-severe	39 (36.45)
GCS *M* (s.d.), *[range]*	14 (11–15)
Cause of injury *n* (%)	
MVA (Car, bicycle, pedestrian)	23 (21.5)
Fall/blow	84 (78.5)
Child function – pre-injury *M* (s.d.), [range]	
Adaptive function (ABAS GAS)	97.0 (15.8), [60–130]
Social skills (ABAS SOC)	101.3 (15.0), [55–130]
Social Participation (CAPS) **	97.9 (5.4), [58.8–100]
Everyday Executive function (BRIEF GEC) ***	48.64 (11.92), [34–97]
Behavior (CBCL Total)	48.3 (10.5), [24–75]
Parent pre-injury function *M* (s.d.), [range]	
Family Function (FAD)	1.6 (0.4), [1.0–2.6]
Parent mental health (GHQ)	1.1 (3.1), [0.0–22.0]

ABAS: GAC, Adaptive Behavior Assessment System Global Adaptive Composite; ABAS:SOC, Adaptive Behavior Assessment System, Social Domain; BRIEF GEC, Behavior Rating Inventory of Executive Function- Global Executive BRIEF GEC, Behavior Rating Inventory of Executive Function- Global Executive Composite; CAPS, Child and Adolescent Participation Scale; CBCL, Child Behavior Checklist; FAD, Family Assessment Device; GHQ, General Health Questionnaire; MVA, Motor vehicle accident; SES, socio-economic status.

*N = 102, ** *N* = 98, *** *N* = 94.

### Trajectory analysis

**Fig. 2.**
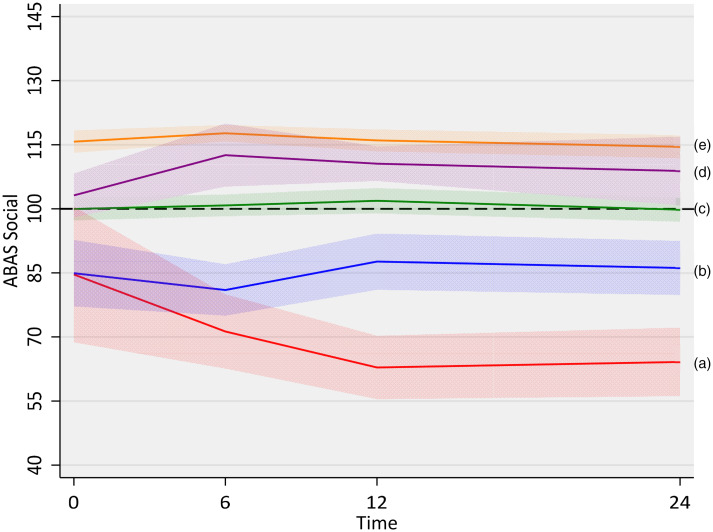
Social trajectories: (a) Impaired (7%); (b): Slow recovery (15%); (c): Intact (45%); (d) Early Recovery (7%); (e) Resilient (39%).

GBTM of social skills from baseline to 2-years post-TBI (ABAS: SOC: T0-T3) was carried out for two to five groups (online Supplementary Table S1). While the four-group solution showed the lowest BIC, this was negligibly different to the 5-group model (difference in BIC = −2.4). The five-group model presented the greatest parsimony (lowest log-likelihood) and entropy > 0.8, while illustrating greater complexity in groups and trajectories. As such, this was retained as the best fitting model. Changes in social skills were evident across the first 12 months post-TBI, but no group demonstrated a significant change from 12- to 24-months (Fig. 2).

- *Impaired* (*n* = 8, 7%): borderline-range pre-injury social skills, deteriorating to impaired by 12 months
- *Slow recovery* (*n* = 16, 15%): borderline pre-injury social skills, a marginal improvement from 6 to 12 months
- *Intact* (*n* = 45, 42%): pre- and post-TBI social skills consistently average
- *Early recovery* (*n* = 7, 7%): early recovery of social skills to 6 months, ongoing average skills.
- *Resilient* (*n* = 31, 29%): pre- and post-TBI social skills consistently above average

Only the Impaired group showed significant deterioration in social skills post-TBI. The Slow Recovery group had low social skills acutely, with a social function returning to pre-injury borderline levels by 12 months, while for the Early Recovery group, social skills improved from pre-injury to 6 months. No significant impact of TBI was identified for Resilient or Intact groups.

### Characteristics of the social trajectory groups

#### Demographic, injury and brain characteristics

Social trajectory membership was not related to demographic characteristics (injury age, sex, SES) or acute clinical indices (severity, GCS, SWI) (Table 3). Examination of MRI variables identified no significant differences across trajectory groups for neural network volumes (DMN, MNEN, SN, CEN, MN). The Slow Recovery group, however, recorded the lowest gray matter volumes across all brain networks examined. For lesion characteristics, no significant differences were identified, despite the Early recovery group having a greater number and volume of SWI lesions.

**Table 3.** Demographic, brain and environmental characteristics of social competence trajectory groups

	A. Impaired (*n* = 8)	B. Slow Recovery (*n* = 16)	C. Intact (*n* = 45)	D. Early Recovery (*n* = 7)	E. Resilient (*n* = 31)	*p*	Pairwise comp
Demographics							
Male, *n* (*%*)	5 (62.5)	11 (68.8)	32 (71.1)	5 (71.4)	21 (67.7)	0.987	
Injury age (yrs), *M* (s.d.)	10 (1.8)	10 (2.8)	10.4 (22.8)	10.5 (2.5)	10.6 (2.1)	0.934	
Severity, *n* (*%*)							
Mild	5 (62.5)	11 (68.8)	25 (55.6)	5 (71.4)	22 (71.0)	0.697	
Moderate/Severe	3 (37.5)	5 (31.3)	20 (44.4)	2 (28.6)	9 (29.0)		
Lowest GCS, *Mdn* (*IQR*)	13.5 (9.5–15.0)	14 (8.5–15.0)	14 (11.0–15.0)	14 (12.0–15.0)	14 (11.0–15)	0.845	
*Brain (neuroimaging)*							
Network volume, *mm*3 (s.d.)							
DMN	116 954.1 (11 477.5)	111 050.3 (12 565.9)	1 164 663.6 (10 806.0)	120 113.6 (12 155.3)	119 033.4 (7833.2)	0.168	
MNEN	98 849.2 (12 965.6)	93 581.3 (11 457.1)	98 061.4 (9659.0)	101 642.9 (11 514.3)	100 760.2 (8000.0)	0.202	
SN	54 981.7 (6155.0)	50 893.6 (4271.2)	53 129.4 (5727.1)	53 852.3 (5967.4)	53 436.3 (3891.1)	0.421	
CEN	176 740.5 (22 009.4)	165 469.3 (16 396.4)	174 014.3 (16 516.6)	179 073.5 (20 480.1)	178 052.7 (12 054.7)	0.147	
MN	149 157.2 (19 076.0)	138 707.6 (16 312.2)	146 173.1 (15 771.6)	156 085.6 (16 320.5)	150 332.7 (10 736.4)	0.067	
SWI							
SWI Vol, Mdn (IQR)	0 (0–0.0)	0 (0–0.0)	0 (0–149.6)	100.5 (0–1698.7)	0 (0–30.7)	0.347	
SWI #, Mdn (IQR)	0 (0–0.0)	0 (0–0.0)	0 (0–3.0)	1.0 (0–13.0)	0 (0–2.0)	0.548	
Environment (SES, family)							
SES, *M* (s.d.)	54.9 (26.3)	54.5 (25.0)	58.7 (21.7)	53.9 (21.6)	60.4 (22.6)	0.888	
FAD GF T0, *M* (s.d.)	1.7 (0.5)	1.8 (0.4)	1.6 (0.3)	1.7 (0.1)	1.5 (0.3)	0.020	b
FAD GF T3, *M* (s.d.)	2.0 (0.2)	1.9 (0.4)	1.7 (0.3)	1.7 (0.3)	1.5 (0.3)	0.005	b,e
GHQ TOT T0, *M* (s.d.)	3.4 (7.7)	0.9 (1.3)	1.5 (3.1)	0.0 (0.0)	0.5 (1.4)	0.110	
GHQ TOT T3, *M* (s.d.)	2.0 (2.4)	0.6 (1.3)	22.3 (3.9)	0.7 (1.9)	1.8 (4.0)	0.604	
FBII T3, M (SD)	0.8 (1.2)	0.2 (0.3)	0.1 (0.4)	0.0 (0.0)	0.1 (0.1)	0.003	b

GCS, Glasgow Coma Score; DMN, Default Mode Network; MNEN, Mirror Neuron Empathy Network; SN, Salience Network, CEN, Central Executive Network; MN, Mentalizing Network; SWI, Susceptibility Weighted Imaging; FAD, Family Assessment Device; FBII, Family Burden of Injury Inventory; GHQ, General Health Questionnaire; GCS, Glasgow Coma Scale; SES, socio-economic status.

a = AvB b = AvC c = AvD d = AvE e = BvC f = BvD g = BvE h = CvD i = CvE j = DvE.

#### Environmental characteristics

SES did not differ across groups, but differences were identified on several proximal environmental factors. For the FAD-GF, baseline and 2-year ratings differentiated the social trajectory groups (*p* = 0.020 and *p* = 0.005 respectively). Pre-injury FAD-GF ratings fell in the Borderline range for all groups, except the Resilient group which was characterized by intact family function at both timepoints. By 2 years, family function had deteriorated to ‘abnormal’ in the Impaired and Slow Recovery groups, while the Early Recovery and Intact groups maintained Borderline function. Pairwise comparisons found a significantly poorer family function for the Slow Recovery compared to the Intact groups, both pre-injury and at 2 years, while by 2 years, the Impaired group FAD-GF ratings had reduced and were poorer than the Early Recovery group.

Parent mental health (GHQ) was in the Borderline range for the Impaired group, but only at baseline, while, in the Intact group, parents reported a decrease in mental health from normal at baseline to Borderline at 2 years. Differences were also detected for the FBII at T3 (*p* = 0.003), with the Impaired group experiencing the greatest family burden (Table 4).

**Table 4.** Cognitive, behavioral and social skills for each social competence trajectory group

	A. Impaired (*n* = 8)	B. Slow Recovery (*n* = 16)	C. Intact (*n* = 45)	D. Early Recovery (*n* = 7)	E. Resilient (*n* = 31)	*p*	Pairwise comp
Child Direct measures							
IQ, *M* (s.d.)	86 (15.7)	88.6 (11.5)	99.1 (11.6)	103.1 (23.1)	102.6 (11.8)	0.001	b,e
Attention/Executive Function, *M* (s.d.)							
Processing Speed Index (PSI)	92.6 (17.3)	94.7 (11.3)	100.6 (13.8)	101.6 (12.5)	102.0 (11.8)	0.213	
Digit Span Test (DST)	7.1 (2.1)	8.9 (3.2)	9.9 (3.0)	10.6 (4.0)	9.3 (2.6)	0.147	
Creature Counting (CCC)	8.3 (4.8)	9.8 (3.2)	10.1 (3.3)	9.6 (4.0)	10.5 (3.0)	0.623	
Walk Don't Walk (WDW)	6.6 (3.6)	6.0 (3.7)	8.0 (3.4)	8.1 (3.9)	7.9 (3.5)	0.301	
Sky Search Dual Task (SSDT)	4.4 (3.7)	6.5 (4.1)	7.2 (3.6)	7.3 (2.1)	6.8 (2.9)	0.406	
Social Cognition, *M* (s.d.)							
Emotional & Emotive faces (EEFT: LOOK)	11.5 (3.0)	11.3 (4.9)	11.43(4.0)	10.4 (4.3)	12.7 (3.6)	0.528	b
Emotional & Emotive faces (EEFT: FEEL)	4.7 (1.9)	5.7 (1.2)	6.2 (2.0)	6.4 (1.7)	7.0 (1.4)	0.022	
Making Inferences (MI)	6.9 (1.8)	6.4 (2.7)	7.7 (2.6)	7.4 (2.3)	8.3 (3.0)	0.218	
*Parent rated measures*							
Adaptive Behavior Assessment, *M* (s.d.)							
ABAS GAC T0	81.5 (14.5)	82.1 (17.6)	95.1 (10.3)	101.7 (9.9)	110.4 (11.6)	<0.001	b,c,d,e,f,g,h
ABAS GAC T1	70.1 (14.9)	82.1 (11.4)	96.1 (9.1)	109.1 (11.9)	110.9 (10.6)	<0.001	b,c,d,e,f,g,h
ABAS SOC T0	84.6 (19.0)	84.9 (14.6)	100.0 (8.9)	103.1 (5.6)	115.7 (7.2)	<0.001	b,c,d,e,f,g,h
ABAS SOC T1	71.3 (9.4)	81.0 (10.9)	100.8 (7.9)	112.6 (8.0)	117.7 (5.1)	<0.001	b,c,d,e,f,g,h
Child Behavior Checklist (CBCL), *M* (s.d.)							
CBCL Total T0	56.5 (15.6)	54.4 (6.7)	49.5 (9.5)	45.3 (6.8)	41.9 (9.0)	<0.001	b,e,h
CBCL Internalizing T0	52.4 (15.0)	52.1 (8.3)	50.7 (10.9)	48.7 (9.3)	43.1 (8.9)	0.012	h
CBCL Externalizing T0	57.6 (13.6)	55.5 (9.9)	49.2 (9.3)	46.4 (7.0)	43.0 (8.0)	<0.001	b,e,h
CBCL Total T3	61.0 (11.6)	52.5 (13.2)	47.9 (11.0)	41.7 (5.4)	40.6 (12.4)	0.001	b,e
CBCL Internalizing T3	53.2 (15.1)	48.2 (11.6)	48.0 (10.4)	43.5 (7.0)	43.0 (9.8)	0.162	b,c,d,e
CBCL Externalizing T3	61.7(11.4)	54.6 (10.7)	48.1 (9.2)	43.3 (4.1)	41.8 (9.7)	<0.001	
Executive function: BRIEF T3, *M* (s.d.)	64.6 (15.3)	56.2 (11.8)	48.1 (9.8)	43.6 (7.7)	45.2 (9.1)	<0.001	b,c,d,e
Social Participation: CASP T3, *M* (s.d.)	90.6 (14.0)	96.6 (5.1)	98.2 (6.2)	99.3 (1.9)	99.3 (1.8)	0.018	b,c

ABAS, GAC, Global Adaptive Composite; ABAS, SOC, Social Domain; BRIEF GEC, Behavior Rating Inventory of Executive Function; CASP, Child and Adolescent Scale of Participation.

a = AvB b = AvC c = AvD d = AvE e = BvC f = BvD g = BvE h = CvD i = CvE j = DvE.

#### Child cognitive function at 2 years

Intellectual ability (IQ) differentiated social recovery trajectories (*p* = 0.001), with the Impaired and Slow Recovery groups having poorer IQ (86.0 and 88.6 respectively) than other groups. Pairwise comparison indicated statistically significant differences between the Impaired and Early Recovery groups and the Slow Recovery and Intact groups. On the BRIEF (everyday executive function, *p* = 0.001), only the Impaired group had scores in the abnormal range, with pairwise comparisons identifying differences between this group and the Early Recovery, Intact and Resilient groups.

For attention/executive function measures, all trajectory groups recorded Borderline to Abnormal scores for SSDT (divided attention), with WDW scores (inhibitory control) also shifted to the lower end of expectations (scaled score range: 6.0–8.1). Only the Impaired group had a score in the Borderline range for DST (attentional capacity). Mean scores for pragmatic language (MI) were Borderline or Abnormal for all trajectory groups. There were no significant trajectory group differences for EEFT: LOOK, with the 5 groups performing similarly and within 1 s.d. of the study control group. In contrast, for EEFT: FEEL, Impaired and Slow Recovery groups recorded the poorest results (*p* = 0.022).

#### Child socio-emotional function

Parent ratings of global adaptive abilities (ABAS: GAC) differentiated trajectory groups (*ps* < 0.001). The Impaired and Slow Recovery groups rated as ‘Abnormal’ both pre-injury and at 6 months, with the Impaired group showing a significant drop in abilities post-injury (11.4 points). For Normal, Early Recovery and Resilient groups, parent ratings were consistently ‘Average’, and significantly higher than ratings of the Impaired and Slow Recovery groups.

Pre-injury and 2-year ratings of child behavior were each associated with trajectory group membership (pre-injury: CBCL: TOT, EXT, *ps*<0.001, INT: *p* = 0.012; 2 years: CBCL:TOT, EXT *p*s ⩽ 0.001). Pre-injury ratings fell within the normal average for all trajectory groups at both timepoints, with the exception that the Impaired group had marginally elevated total and externalizing behavior symptoms pre-injury, which increased to behaviors of concerns by 2 years (CBCL:TOT: 56.5 *v.* 61.0; CBCL:EXT: 57.6 *v.* 61.7). Of note, the Resilient group were rated to have very few internalizing or externalizing symptoms at any timepoint.

Social participation (CASP) at 2 years was significantly different across trajectory groups (*p* = 0.018), with the Impaired and Slow Recovery groups recording abnormally low levels of social participation and the Intact group falling in the Borderline range.

## Discussion

Pediatric TBI is commonly considered to have a significant impact on social function, with social impairment a persisting and debilitating consequence of injury. However, the incidence and degree of these problems, and their underlying mechanisms are poorly documented. In this study, we found that a significant proportion of children experience social impairment post-TBI. For this group, problems are apparent in the early stages of recovery, plateau by 12 months and persist to at least 2 years post-injury. We identified five distinct social recovery trajectories. Where social problems were detected, they were characterized by a complex interplay between environment pre- and post-TBI child and family factors, with injury factors playing a lesser role. Social resilience was linked to intact family and parent function, better pre-injury adaptive abilities, post-TBI cognition and social participation. In contrast, vulnerability in the social domain was related to poorer pre- and post-injury adaptive abilities, greater behavioral concerns, and poorer pre- and post-injury parent mental health and family function.

### Social impairment and specific social recovery trajectories over the 2 years post-TBI

Overall, our findings suggest that persisting social impairment is present in approximately 1 in 4 children after TBI, with 77.5% of our sample having average social skills by 2 years post-TBI. Of the five social recovery trajectories identified, three (Early Recovery, Intact, Resilient) were characterized by largely intact social skills and only two trajectory groups (Impaired, Slow Recovery), constituting 22.5% of the study population, exhibited persisting social impairments. Where detected, soicial skills recovery or deterioration were evident in the first 12-months post-injury, with no significant changes from 12 to 24-months post-injury. For 71% of children (*n* = 76, Resilient, Intact groups) we found no evidence that TBI impacted social skills, with function in the average range across the two-year study period, from pre-injury to 2 years post-TBI.

### Risk and resilience factors for social recovery and persistent impairment

Sex and age at injury were not associated with social recovery trajectories, nor were GCS or injury severity. We examined two aspects of brain structure, volumetric MRI of several large-scale neural networks and volume and number SWI lesions, reflecting deposits of blood in brain tissue. No statistically significant brain structure differences were identified across the trajectory groups. With respect to the environment, SES was not related to trajectory membership, however, proximal factors were, with pre-injury and 2-year family function, and 2-year perceived family burden discriminating trajectory groups. For family function, all trajectories were characterized by Borderline or Abnormal pre- and post-TBI family function, except in the Resilient group.

Direct measures of child cognition were associated with few group differences. For IQ, the Impaired and Slow Recovery groups demonstrated borderline-range abilities. Supporting the significant literature showing that pediatric TBI is associated with deficits in more demanding cognitive domains, on tasks tapping specific higher-order cognitive skills (divided attention, inhibitory control) and social cognition (pragmatic language, theory of mind) while no trajectory specific patterns were identified, all groups recorded depressed performances. Parent ratings of their child's function in everyday life (adaptive abilities, social skills, behavior, social participation) were consistently different across trajectories, with a visual inspection of findings suggesting that the Impaired and Slow Recovery groups were largely responsible for these differences.

### Characterizing social recovery trajectories

The three trajectories characterized by intact social skills, not surprisingly, demonstrated few risk factors. The ***Resilient trajectory*** was overall unremarkable. In keeping with consistently above-average social skills post-TBI, it included relatively few children with moderate/severe TBI. Brain and environmental indices were normal as were adaptive skills and social participation. Behavioral symptoms were few and most cognitive abilities were intact. The only lower results were for higher-order cognition (divided attention, inhibitory control, pragmatic language). The ***Intact trajectory*** was also largely resilient to the challenges associated with child TBI. This group demonstrated average social skills across the duration of the study, apparently unaffected by the impact of TBI. While the group included more moderate/severe injuries than any other group, brain metrics were normal. Proximal environmental risks were present, with borderline parent mental health at 2 years. Elevated family function scores were evident pre-TBI and at 2 years, however scores were stable across time, suggesting no TBI-related increase in family dysfunction. Adaptive skills, behavior, social participation and most cognitive abilities were intact, with lower results only for higher-order cognition (divided attention, pragmatic language). The ***Early Recovery trajectory*** demonstrated relatively lower pre-injury social skills, which improved to upper-average levels by 6 months post-TBI. Approximately one-quarter of group members had sustained a moderate-severe TBI, and, while brain volumes did not significantly differ from other trajectory groups, there was evidence of SWI lesions, which may explain initially lower levels of social function. These lesions are reported to resolve post-acutely, possibly explaining the rapid improvement in social skills in the 6 months post-injury. Pre-TBI and 2-year family function were borderline, but stable. Child variables (direct and parent-rated) were consistent with those described for the Intact group.

The Impaired and Slow Recovery trajectories demonstrated more wide-reaching risk factors, with pre-injury data suggesting that some post-TBI impairment may be explained by pre-existing vulnerabilities in child function. The ***Impaired Trajectory*** was the only group to display deteriorating social skills from pre-injury to 12 months, after which social skills stabilized, with impairments persisting. Compared to other groups, the Impaired group demonstrated multiple significant risk factors. As for other trajectories, brain imaging variables were unremarkable, but 3/5 participants sustained a moderate/severe TBI. Family function pre-injury was borderline, reducing to abnormal by 2 years, parent mental health was within the borderline range, and the perceived burden was high. Further, this group was characterized by pre-injury impairments in adaptive and social skills, and borderline externalizing behaviors which had escalated to abnormal by 2 years. In addition, at 2 years, cognitive skills, social participation and everyday executive skills were all abnormal. Lastly, the ***Slow Recovery trajectory*** also displayed vulnerability post-TBI, with low social skills pre-injury, dipping slightly post-injury before returning to pre-injury levels by 12 months post-injury. Compared to other trajectory groups, brain volumes were lower across all brain networks. While similar to the Impaired trajectory, deficits tended to be of a lesser degree across the family environmental indices, pre-injury adaptive skills, cognition and social participation. Behavior and everyday executive skills were normal in this group.

While the prospective longitudinal design of our study, and our high retention rates, allowed us to plot social recovery, and its pre- and post-TBI correlates, over time, results need to be interpreted in the context of some limitations. Firstly, we chose to focus on everyday social skills as our primary social outcome, to maximize ecological validity and address parent concerns. As a consequence, our capacity to target specific social cognitive skills and their brain correlates was limited, which may partly explain the lack of relationship we identified between brain structure and social impairment. Additionally, due to our inability to assess white matter microstructure in our sample, we may have missed an important injury-related factor. Further studies of larger TBI samples are needed to evaluate whether abnormalities of white matter microstructure may explain additional variance in post-injury social outcomes.

In keeping with our aims, we relied on parent-report for much of our data. There is surprisingly little acknowledgement (or exploration) in the literature of the limitations of using such ratings, despite the established bidirectionality of parent-child relationships and perceptions. In the TBI field, where brain injuries may impair children's awareness of their difficulties, and parents may be highly concerned, interpretation of findings based on parent responders should be made cautiously. We acknowledge the lack of robust, developmentally appropriate measures available to assess children's social skills is problematic, which may have resulted in an under-estimation of rates of social impairment in our sample. The development of more robust tools is needed to better characterize deficits in the social domain. Finally, our analyses have generated small group sizes, and may have resulted in a loss of power.

Despite these limitations, this study has both theoretical and clinical relevance. Findings confirm the importance of employing a biopsychosocial framework, such as the SOCIAL model, to understand the factors that underpin post-injury social impairments and to guide the development of interventions to optimise post-injury social outcomes. Our results highlight the bi-directional relationship between the child and their family following TBI, and confirm the key role of the child's environment, and the well-being of the family in particular, for optimal outcome. Importantly, as demonstrated in previous research (McMillan et al., 2020; Muscara et al., 2018; Wade et al., 2011; Woods, Catroppa, Eren, Godfrey, & Anderson Woods, 2013), family factors (e.g., family function, parent mental health, perceived burden) may represent modifiable risk factors. Their identification and assessment within a family-centred model of care can guide effective intervention and treatment in the months and years after TBI, resulting in optimal social competence.

## Conclusions

Our findings suggest that one in four children with TBI experience social impairment, which emerges post-injury and persists for at least 2 years. Within our sample, we identified five distinct social recovery profiles, which were characterized by a complex interplay between proximal environmental and pre- and post-TBI child factors, with injury and distal environmental factors playing a lesser role. Deficits in higher-order cognitive skills, including attention, executive function, and social communication were common across all groups, and could be potential targets for assessment, monitoring and treatment following child TBI.

Resilience was linked to intact family and parent function, better pre- injury adaptive abilities, post-TBI cognition and social participation. In contrast, vulnerability in the social domain was related to poorer pre- and post-injury adaptive abilities, greater behavioral concerns, and poorer pre- and post-injury parent mental health and family function. Attention to these ‘modifiable risk factors’ through the delivery of evidence-based interventions offers the opportunity to enhance recovery of social competence.

## Supplementary material

For supplementary material accompanying this paper visit https://doi.org/10.1017/S0033291722000186. click here to view supplementary material
