# Supplementary material for: What predicts persisting social impairment following pediatric traumatic brain injury: contribution of a biopsychosocial approach
Source: Psychol Med. 2022 Feb 22;53(8):3568–79. doi: 10.1017/S0033291722000186 (PMC10277758; doi:10.1017/S0033291722000186)
Supplement: Supplementary file 1 [file S0033291722000186sup001.docx]

**Supplementary Table 1. Group-based trajectory model fit statistics for two to five group models.**

| *k* | Trajectories^1^ | Log-likelihood | AIC^2^ | BIC^3^ | Entropy |
| --- | --- | --- | --- | --- | --- |
| 3 | 0 0 0 | -1464.62 | -1470.62 | -1478.64 | 0.75 |
| 4 | 0 0 0 0 | -1458.48 | -1466.48 | -1477.17 | 0.76 |
| 5 | 2 0 0 0 0 | -1451.51 | -1463.51 | -1479.55 | 0.82 |

*k* = Number of groups
^1^ Listed as order of powers (0 = intercept, 1 = linear, 2= quadratic, 3=cubic)
^2^ AIC: Akaike Information Criterion
^3^ BIC: Bayesian Information Criterion
